# Impact of romosozumab treatment for 24 months after switching from denosumab: three case reports

**DOI:** 10.1093/jbmrpl/ziaf070

**Published:** 2025-04-23

**Authors:** Seong Hee Ahn, Seung-Eun Lee, Seung Hun Lee, Ha Young Kim, Seongbin Hong, Jung-Min Koh

**Affiliations:** Division of Endocrinology and Metabolism, Department of Internal Medicine, Inha University Hospital, Inha University School of Medicine, Incheon 22332, South Korea; Department of Internal Medicine, Ulsan University Hospital, University of Ulsan College of Medicine, Ulsan 44033, South Korea; Division of Endocrinology and Metabolism, Department of Internal Medicine, Asan Medical Center, University of Ulsan College of Medicine, Seoul 05505, South Korea; Division of Endocrinology, Department of Internal Medicine, Gangneung Asan Hospital, University of Ulsan College of Medicine, Gangneung 25440, South Korea; Division of Endocrinology and Metabolism, Department of Internal Medicine, Inha University Hospital, Inha University School of Medicine, Incheon 22332, South Korea; Division of Endocrinology and Metabolism, Department of Internal Medicine, Asan Medical Center, University of Ulsan College of Medicine, Seoul 05505, South Korea

**Keywords:** bone turnover markers, denosumab, romosozumab, sequential treatment

## Abstract

Prior denosumab use in osteoporosis patients diminishes the BMD gains from a subsequent 12-mo romosozumab regimen. However, few studies have explored BMD gains after 24 mo of romosozumab therapy. BMD at the LS-BMD, FN-BMD, and TH-BMD and P1NP and C-terminal telopeptide of type I collagen (CTX) levels were measured after 24 mo of romosozumab therapy in 3 osteoporosis cases with prior denosumab use. The changes in cases 1-3 at 12 and 24 mo, respectively, were as follows: LS-BMD (−2.9% and 7.4%, 0.9% and 7.9%, 7.2% and 7.9%), FN-BMD (3.2% and 7.1%, −1.2% and 2.0%, 2.7% and 2.1%), and TH-BMD (−1.2% and 6.7%, −4.9% and −0.3%, −0.3% and 3.2%). The P1NP and CTX levels in case 1 (18.80 ng/mL, 0.042 ng/mL) peaked at 6 mo (185.00 ng/mL, 1.280 ng/mL) and then decreased at 24 mo by 60.8% and 65.3%, respectively. The P1NP and CTX levels in case 2 (17.10 ng/mL, 0.059 ng/mL) peaked at 12 mo (132.00 ng/mL, 1.190 ng/mL) and then decreased at 24 mo by 65.3% and 24.3%, respectively. In case 3, the P1NP and CTX levels (56.10 ng/mL, 0.490 ng/mL) increased at 24 mo by 233.3% and 375.5%, respectively. This is the first report of a 24-mo romosozumab regimen in a small group of osteoporosis patients with prior denosumab use having varied effects on BMD and bone turnover. Nonetheless, larger controlled studies are needed to confirm these findings.

## Introduction

Romosozumab (Romo) has dual osteoanabolic and antiresorptive effects, resulting in substantial BMD gains and significant fracture risk reduction.^1–3^ Consequently, Romo is recommended as first-line therapy for patients at very high fracture risk and second-line therapy for patients with severe osteoporosis and persistently low *T*-scores or fracture despite previous denosumab (Dmab) therapy.^4^^,^^5^

As first-line therapy, Romo yields the greatest BMD gains (LS-BMD: 13.7%-18.2%, FN-BMD: 4.9%-5.1%, TH-BMD: 3.0%-6.2%);^6–10^ however, prior Dmab therapy attenuates Romo-induced BMD gains (LS-BMD: 5.3%-7.9%, FN-BMD: 0.7%, TH-BMD: −1.0%-0.9%).^6–11^ Two potential explanations include reduced early anabolic effects of Romo and insufficient antiresorptive effect to suppress rebound increase in osteoclast activity following Dmab withdrawal.^12^ Supporting this, C-terminal telopeptide of type I collagen (CTX) levels increased after switching from long-term Dmab to 12-mo Romo therapy.^9^^,^^10^^,^^13^

In a related context, switching from Dmab to 24 mo of teriparatide (TPTD), another osteoanabolic agent, initially decreased LS-BMD during the first 6 mo, followed by an 18-mo increase, yielding a net 4.8% BMD gain.^14^ This suggests that extending Romo therapy to 24 mo may similarly enhance BMD gains. We thus compared BMD changes and bone turnover marker (BTM; P1NP and CTX) levels after 12- and 24-mo Romo therapy in patients previously treated with Dmab.

### Case presentations

The study was approved by the Institutional Review Board of Asan Medical Center (No: 2023-0429) and conducted in compliance with the Declaration of Helsinki. Due to its retrospective design, informed consent was waived.

The same dual-energy X-ray absorptiometry equipment (Lunar Prodigy) was used for all BMD measurements, with coefficients of variation (CVs) of 0.82%, 1.08%, and 1.06% for LS-BMD, FN-BMD, and TH-BMD, respectively. BTM levels were measured after at least 8 hr of fasting using a chemical-luminescence immunoassay (Roche Diagnostics), with intra- and inter-assay CVs of 1.0%-2.7% and 1.6%-2.7% for CTX and 2.0%-2.1% and 2.7%-3.8% for P1NP.

**Figure 1:**
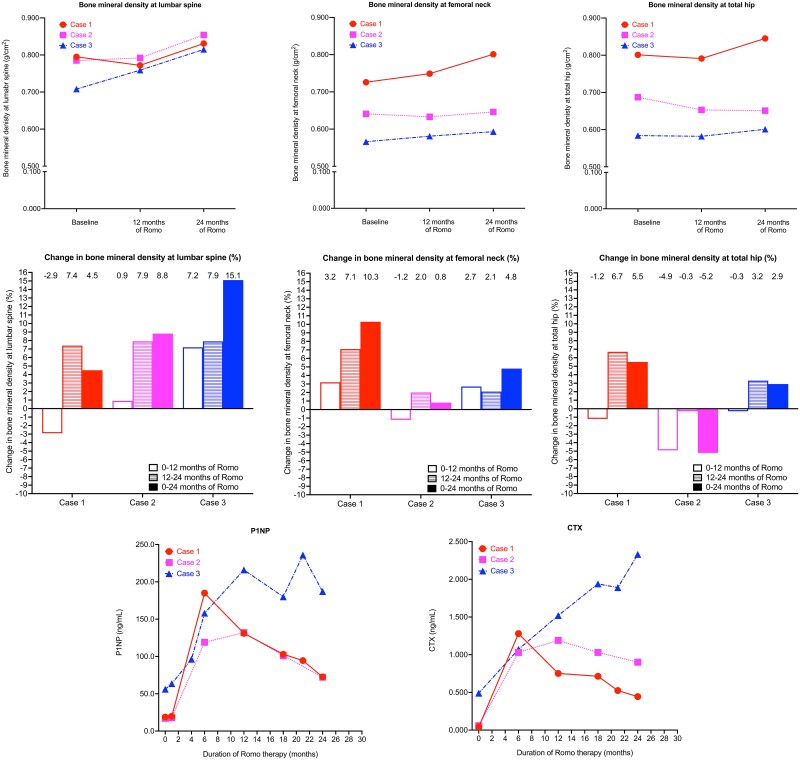
BMD and BTM values were measured at 12 and 24 mo in the 3 study cases. Abbreviations: BMD, bone mineral density; BTM, bone turnover marker; CTX, C-terminal telopeptide of type 1 collagen; P1NP, procollagen type 1 N-propeptide.

Changes in LS-BMD, FN-BMD, and TH-BMD at 12 and 24 mo were −2.9% and 7.4% (Case 1), 0.9% and 7.9% (Case 2), and 7.2% and 7.9% (Case 3); 3.2% and 7.1% (Case 1), −1.2% and 2.0% (Case 2), and 2.7% and 2.1% (Case 3); and −1.2% and 6.7% (Case 1), −4.9% and −0.3% (Case 2), and −0.3% and 3.2% (Case 3), respectively (Figure 1). P1NP remained within the reference range at baseline (Table 1) and 1 mo in Cases 1 and 2, peaked at 6 mo in Case 1 and 12 mo in Case 2, and declined at 24-mo in both cases. CTX was suppressed at baseline in Cases 1 and 2, peaked at 6 mo in Case 1 and 12 mo in Case 2, with steep and gradual declines by 24 mo, respectively. Case 3 showed persistently elevated P1NP and CTX over 24 mo, with higher baseline levels than those of Cases 1 and 2.

**Table 1:** Patient characteristics.

	Case 1	Case 2	Case 3
**Age at Romo commencement (yr)**	57	56	72
**Height (cm)**	160.4	154.7	155.6
**Weight (kg)**	50.0	40.9	46.7
**BMI (kg/m^2^)**	19.43	17.09	19.31
**Prevalent fractures**	None	None	None
**Other drugs administered prior to Dmab**	Risedronate for 2 yr	None	None
**Prior Dmab doses (n)**	8	6	3
**BMD values prior to the last Dmab dose**			
**LS-BMD (g/cm^2^)/*T*-score at LS**	0.794/−2.8	0.774/−3.2	0.697/−3.9
**FN-BMD (g/cm^2^)/*T*-score at FN**	0.733/−1.8	0.648/−2.5	0.559/−3.4
**TH-BMD (g/cm^2^)/*T*-score at TH**	0.805/−1.4	0.680/−2.5	0.578/−3.5
**Pre-Romo BMD values**			
**LS-BMD (g/cm^2^)/*T*-score at LS**	0.795/−2.8	0.785/−3.2	0.708/−3.9
**FN-BMD (g/cm^2^)/*T*-score at FN**	0.726/−1.8	0.641/−2.5	0.566/−3.4
**TH-BMD (g/cm^2^)/*T*-score at TH**	0.801/−1.4	0.687/−2.5	0.584/−3.5
**Corrected serum calcium (mg/dL)**	9.7	9.0	9.2
**eGFR (mL/min/1.73 m^2^)**	92	97	72
**Pre-Romo BTM levels**			
**P1NP**	18.80	17.10	56.10
**CTX**	0.042	0.059	0.490
**25(OH)D (ng/mL)**			
**Pre-Romo**	44.6	27.5	32.4
**After 12 mo of Romo**	48.6	35.0	38.6
**After 24 mo of Romo**	40.5	32.3	35.5
**Adverse events during Romo therapy**			
**Incidental vertebral fracture**	None	None	None
**Cardiovascular event**	None	None	None

The reference P1NP level range is 16.27-73.87 ng/mL in postmenopausal women and 15.13-58.59 ng/mL in premenopausal women. The reference CTX level range is 0.177-1.015 ng/mL in postmenopausal women and 0.136-0.689 ng/mL in premenopausal women.

Abbreviations: BMD, bone mineral density; BTM, bone turnover marker; CTX, C-terminal telopeptide of type 1 collagen; eGFR, estimated glomerular filtration rate; FN-BMD, BMD at FN; LS-BMD, BMD at lumbar spine, P1NP, procollagen type 1 N-propeptide; TH-BMD, BMD at TH; 25(OH)D, 25-hydroxycholecalciferol.

All patients received vitamin D supplementation. Case 2 had vitamin D insufficiency (<30 ng/mL) at baseline but adequate levels (32.5 ng/mL) after 1 mo of Romo therapy. Over 24 mo of Romo therapy, no adverse events, including vertebral fractures or cardiovascular events, were observed at monthly follow-ups.

## Discussion

Despite including only 3 cases, this is the first report of BMD and BTM changes following a switch from Dmab to 24-mo Romo therapy. Previous studies evaluated the outcomes of only 12-mo of Romo after Dmab and reported increases in BTMs till or above baseline levels and no significant hip BMD gains.^6–11^^,^^13^ However, the 24-mo Romo therapy in our study resulted in peaked and decreased BTMs in 2 cases, greater LS-BMD gains during 12-24 mo than 0-12 mo in all cases, and significant hip BMD gains in 2 cases. LS-BMD gains were the greatest in Case 3 (highest baseline P1NP and continuous increase over 24 mo), followed by Case 2 (later P1NP peak at 12 mo) and Case 1 (earlier P1NP peak at 6 mo). Case 1 had an earlier BTM peak at 6 mo, a steeper CTX decline (65.3% at 24 mo), and the highest hip BMD gains. Overall, 24-mo Romo therapy had varied effects on BMD and BTMs.

Given the small sample size, the exact mechanisms underlying BMD changes over 12-24 mo compared to 0-12 mo Romo therapy remain unclear. However, despite the known limitations in interpreting BTM levels, we identified 2 BTM patterns, possibly influenced by prior Dmab duration (<2 yr vs ≥2 yr), that may explain the observed BMD changes.

First, Case 3 (<2 yr of Dmab) had the highest baseline P1NP level, which continuously increased over 24 mo. Most bone formation with Romo is modeling-based,^15^ but some initial anabolic effects may be related to remodeling overfilling.^16^ Although drug-naïve patients typically have a rapid P1NP peak at 14 d followed by a gradual decline,^6^^,^^17^ delayed P1NP increases at 6 mo after prior Dmab use suggest attenuated early anabolic effects of Romo due to lower bone remodeling surface^16^ or rebound increased bone resorption after Dmab cessation.^12^ However, despite this delay, continuous P1NP increase led to the highest LS-BMD gains at 24 mo and comparable LS-BMD and FN-BMD gains and a TH-BMD increase of 3.2% over 12-24 mo versus a −0.3% loss over 0-12 mo, suggesting that sustained anabolic effects contributed to BMD gains in Case 3.

Second, P1NP peaked at 6 and 12 mo in Cases 1 and 2 (≥2 yr of Dmab), respectively, followed by a steeper CTX decline at 12 mo in Case 1 and a slower CTX decline at 24 mo in Case 2. Progressive CTX increase during the 12-mo Romo therapy suggests insufficient antiresorptive potency to fully suppress bone turnover rebound after Dmab cessation, resulting in increased risk of multiple vertebral fractures^12^ and some BMD loss over 0-12 mo and persistent TH-BMD loss in Case 2 over both 0-12 (−4.9%) and 12-24 mo (−0.3%). However, BMD gains at all sites in Case 1 and in LS-BMD and FN-BMD in Case 2 were greater over 12-24 mo than over 0-12 mo, while TH-BMD loss in Case 2 was lower over 12-24 mo. These trends align with findings for zoledronic acid use after Dmab cessation,^18^^,^^19^ where greater 12-24 mo BMD gains suggested a diminished Dmab withdrawal-related rebound bone resorption effect over 12-24 mo than 0-12 mo. In Case 1, the early 6-mo P1NP peak and steep 24-mo CTX decline widened the anabolic window, resulting in the greatest FN-BMD (10.3%) and TH-BMD (5.5%) gains. This supports previous reports of early anabolic window widening due to Romo improving treatment response.^20^ A novel overlapping treatment strategy, where Romo therapy began 3 mo after the last Dmab dose, and Dmab therapy was resumed 6 mo after Romo to suppress CTX increase and widen the anabolic window, yielded greater LS-BMD and TH-BMD gains compared with the standard Dmab-to-12 mo Romo switch.^12^ Thus, strategies to optimize the anabolic effects of Romo that precede and suppress rebound bone resorption might maximize BMD gains after Dmab treatment.

In conclusion, this first report of the effects of 24-mo Romo therapy with prior Dmab use demonstrates that longer treatment produces differential and beneficial BMD gains and dynamic BTM changes. Larger controlled studies are needed to confirm these findings.

## Data Availability

The data that support the findings of this study are available from the corresponding author upon reasonable request.
